# The clinical impact of macrophage polarity after Kasai portoenterostomy in biliary atresia

**DOI:** 10.3389/fped.2024.1338131

**Published:** 2024-01-22

**Authors:** Kazuya Nagayabu, Shigehisa Fumino, Ai Shimamura, Yuki Sengoku, Mayumi Higashi, Masafumi Iguchi, Shigeyoshi Aoi, Shibata Saya, Maki Hirai, Hiroshi Ogi, Aya Miyagawa-Hayashino, Eiichi Konishi, Kyoko Itoh, Tatsuro Tajiri, Shigeru Ono

**Affiliations:** ^1^ Department of Pediatric Surgery, Kyoto Prefectural University of Medicine, Kyoto, Japan; ^2^ Department of Gastroenterological & Pediatric Surgery, Gifu University, Gifu, Japan; ^3^ SCREEN Holdings Co., Ltd., Kyoto, Japan; ^4^ Department of Pathology and Applied Neurobiology, Graduate School of Medical Science, Kyoto Prefectural University of Medicine, Kyoto, Japan; ^5^ Department of Surgical Pathology, Kyoto Prefectural University of Medicine, Kyoto, Japan; ^6^ Department of Pediatric Surgery, Faculty of Medical Science, Kyushu University, Fukuoka, Japan

**Keywords:** biliary atresia, macrophage polarity, postoperative cholangitis, native liver survival rate, multiple immunohistochemistry

## Abstract

**Introduction:**

Biliary atresia (BA) is a cholestatic hepatopathy caused by fibrosing destruction of intrahepatic and extrahepatic bile ducts, and its etiology has not been clearly revealed. In BA, liver fibrosis progression is often observed even after Kasai portoenterostomy (KPE), and more than half of cases require liver transplantation in their lifetime in Japan. Macrophages play an important role in liver fibrosis progression and are classically divided into proinflammatory (M1) and fibrotic macrophages (M2), whose phenotypic transformation is called “macrophage polarity.” The polarity has been reported to reflect the tissue microenvironment. In this study, we examined the relationship between macrophage polarity and the post-KPE clinical course.

**Materials and methods:**

Thirty BA patients who underwent KPE in our institution from 2000 to 2020 were recruited. Multiple immunostainings for CD68, CD163, CK19, and α-SMA were carried out on liver biopsy specimens obtained at KPE. ROC curves were calculated based on each clinical event, and the correlation with the clinical data was analyzed.

**Results and discussion:**

The M2 ratio, defined as the proportion of M2 macrophages (CD163-positive cells), was correlated inversely with the occurrence of postoperative cholangitis (AUC: 0.7602). The patients were classified into M2 high (*n* = 19) and non-high (*n* = 11) groups based on an M2 ratio value obtained from the Youden index ( = 0.918). As a result, pathological evaluations (Metavir score, αSMA area fraction, and CK19 area fraction) were not significantly different between these groups. In mild liver fibrosis cases (Metavir score = 0–2), the M2 non-high group had a significantly lower native liver survival rate than the high group (*p *= 0.02). Moreover, 4 out of 8 cases in the M2 non-high group underwent early liver transplantation within 2 years after KPE.

**Conclusions:**

Non-M2 macrophages, including M1 macrophages, may be correlated with postoperative cholangitis, and the M2 non-high group in mild liver fibrosis cases had a significantly lower native liver survival rate than the high group, requiring early liver transplantation in this study. Preventing advanced liver fibrosis is a key factor in improving native liver survival for BA patients, and liver macrophages may play important roles in liver homeostasis and the promotion of inflammation and fibrosis.

## 1 Introduction

Biliary atresia (BA) is a cholestatic hepatopathy caused by fibrosing destruction of intrahepatic and extrahepatic bile ducts. If untreated, the liver develops fatal cirrhosis, and even after Kasai portoenterostomy (KPE), approximately half of the patients need liver transplantation because of the progression of cirrhosis within 10 years after KPE. Therefore, prevention of liver fibrosis progression after KPE in BA is the greatest challenge for paediatric surgeons. Although various immune responses have been implicated in liver fibrosis, the details of the etiology and the molecular mechanism of progression remain unclear (1, 2).

The cellular mechanism of liver fibrosis is that stellate cells are activated to become fibroblasts and deposit extracellular matrix in the liver (3). Among the multiple factors that activate stellate cells, we concentrated on liver macrophages in this study. Macrophages are classically divided into two categories: proinflammatory macrophages (M1) and fibrotic macrophages (M2) (4). M1 macrophages have high antigen-presenting activity and drive type 1 T helper (Th1) cells into the tissue as part of the innate and adaptive immune response. On the other hand, M2 macrophages are activated by Th2 cells and are helpful not only for tissue reconstruction but also for immune regulation as part of the anti-inflammatory response (5). M1 can change to M2 and vice versa, altered by various factors such as microorganisms, tissue microenvironment, and cytokine signals. This phenotypic transformation is called “macrophage polarity”, reflecting the surrounding microenvironment, and there have been few reports on macrophage polarity in BA (4).

In this study, we studied macrophage polarity in liver biopsy specimens of BA with a novel multiple immunohistochemistry (IHC) method and evaluated its clinical impact on the post-KPE clinical course in BA.

## 2 Materials and methods

### 2.1 Patients

From 2008 to 2020, thirty patients with BA were consecutively treated at our institute. All patients underwent KPE during early infancy, and liver tissue was obtained by wedge resection from the liver edge at the same time. The patients were administered steroids after surgery until jaundice subsided. As control specimens, five non-BA cases who underwent liver biopsy within 3 months of birth were recruited. Their clinical diagnosis included two cases of neonatal hepatitis, two cases of congenital biliary dilatation, and one case of 2,3-β-hydroxy steroid oxidoreductase deficiency.

#### 2.2.1 Markers for macrophages and liver structures

Several biomarkers are available to distinguish these phenotypes. CD68 is the most common antigen of macrophages, and its antibody was used in pioneering research on BA on inflammatory cells, including macrophages, as reported by M Davenport (6). M2 polarization, which is known to be tumor-associated macrophages, upregulates the mRNA expression levels of CD163, and the antibody for CD163 is commonly used as a highly specific antibody for M2 (7, 8). Therefore, antibodies against CD68 and CD163 were used to distinguish the types of macrophages in this study. Recently, M2 are subdivide into several phenotypes based on the expression profiles of cytokines and chemokines (9). However, the correlation between the phenotypes of M2 macrophage and liver fibrosis have not been elucidated yet. Therefore, we used CD163 which expresses M2 comprehensively according to the past study (4). α-Smooth muscle (αSMA), which is positive for activated myofibroblasts, and cytokeratin 19 (CK19), which is positive in developing bile duct cells, were also used as markers of liver structures (10, 11).

#### 2.2.2 Multiple immunohistochemistry with a single FFPE tissue section

The liver tissues obtained during surgery were immediately fixed in 10% formalin and embedded in paraffin (FFPE). A single 4-μm-thick section was immunohistochemically stained multiple times employing the following primary antibodies: anti-CD68 antibody (PA0273; Leica Biosystems, Nussloch, Germany), anti-CD163 antibody (PA0090; Leica Biosystems), 1:200 anti-CK 19 antibody (ab220193; Abcam, Cambridge, UK), and 1:4,000 anti-α-SMA antibody (1395–1-AP; Proteintech, Rosemont, USA). After deparaffinization, we performed conventional immunostaining by the enzyme antibody method. The alcohol-soluble peroxidase substrate 3-amino-9-ethylcarbazole (AEC) was used as a label, and the sections were scanned as digital images with NanoZoomer S60 (Hamamatsu Photonics K.K., Shizuoka, Japan). After that, the slide was washed in ethanol to decolorize the AEC, and heat-induced epitope retrieval was performed. Activated nonstained sections were obtained again, and immunohistochemistry was repeated with another primary antibody by the above procedure. This antibody-stripping procedure does not affect IHC sensitivity (12).

Sets of serial images obtained after multiple rounds of sequential IHC were aligned semiautomatically with CellProfiler software. The generated images were subsequently linked to ImageJ, and AEC/hematoxylin-color data were extracted by color deconvolution algorithms changing to grayscale. Finally, we could recognize pseudocolored images.

#### 2.2.3 A cell recognition algorithm to quantify the number of each macrophage phenotype

Image preprocessing and region of interest (ROI) selection were performed as follows. Iteratively digitized images were accurately coregistered using in-house software (SCREEN Holdings Co., Ltd.). The software calculated the coordinates of each image relative to a reference image (any one of the images). Using these coordinates, a set of noncompressed TIFF images for each ROI was extracted. AEC color extraction of the single-marker images was performed by the ImageJ plugin Color Deconvolution (www.mecourse.com/landinig/software/software.html) in Fiji Life-Line version 2017-May-30 (https://imagej.net/software/fiji/downloads). Single cell-based segmentation was performed by one kind of convolutional neural network, WGAN (13). FusionNet was utilized as a generator part, and its convolution unit consisted of 45 layers (9 blocks × 5 units) (14). This segmentation system required CD68- or CD163-stained pathological tissue images as input data and produced cell-segmented images. Training data for the segmentation system were composed of digitized images of CD68- or CD163-stained liver images (approximately 140 mm^2^ area), labelled manually. With these training data, cell recognition was performed by AI based on supervised learning. Quantification of staining intensity was performed using in-house software (SCREEN Holdings Co., Ltd.).

According to this algorithm, we defined M2 macrophages as positive for CD163. Cells that were positive for CD68 and negative for CD163 were treated as non-M2 macrophages, including M1 macrophages. The “M2 ratio” was defined as the ratio of M2 macrophages to total macrophages. Most CD163-positive cells were also positive for CD68, but some were negative in our IHC. Considering the high sensitivity and specificity of the CD163 antibody for M2 macrophages (8), CD68-negative and CD163-positive cells were included in M2 macrophages in our study.

#### 2.2.4 Measurement of the positive area fraction from IHC images of α-SMA and Ck19

AEC color extraction of the single-marker images (α-SMA and CK19) was performed by ImageJ plugin (same as above), and the area fraction per portal area traced in the microscopic field of view was calculated. The quantitative values were averaged across 3–5 fields of view per case.

#### 2.2.5 Evaluation of the degree of liver fibrosis

We determined the degree of liver fibrosis using hematoxylin-stained sections according to the Metavir fibrosis score, which is commonly used: 0 = no fibrosis, 1 = fibrous expansion of portal areas, 2 = portal-to-portal fibrosis, 3 = portal-to-portal and portal-to-central fibrosis, or 4 = liver cirrhosis (16).

### 2.3 Statistical analyses

The chi-square test and the Mann–Whitney *U* test were used for comparisons between two groups, the Kruskal–Wallis test was used among the three groups, and the Holm test was added when necessary. All *p* values were two-sided, and *p* values of 0.05 or less were considered statistically significant. Graphs were created using GraphPad Prism9® (GraphPad Software, San Diego, California, USA). The statistical analyses were performed with EZR (Saitama Medical Center, Jichi Medical University, Saitama, Japan), which is a graphical user interface for R (The R Foundation for Statistical Computing, Vienna, Austria) (16).

### 2.4 Ethics approval and consent to participants

All patients were informed about the study and consented to their inclusion. Analyses of this study were approved by the ethics committee of our institution (ERB-C-617).

## 3 Results

### 3.1 Patient background

Table 1 presents the clinical background of patients with BA and non-BA cases. There was no significant difference between these two groups in sex, age at liver biopsy, or preoperative laboratory data except for GGTP (*p *= 0.0496). The median follow-up period for the BA group was 70 (8–170) months. According to the Japanese clinical guidelines for BA, the obstruction site of the extrahepatic bile duct was classified into 3 types (I: common bile duct, II: hepatic duct, and III: hepatic hilum). Ninety percent of the BA patients were categorized as type III, which was a similar tendency to the domestic aggregation in Japan (17).

**Table 1:** Clinical background of patients with biliary atresia and controls.

		Biliary atresia patiets (*n* = 30)		Controls (*n* = 5)		*p-*value
Gender	(M:F)	10:20		2:3		1
Age at liver biopsy	(day)	60	(31–93)^a^	47	(14–75)^a^	0.345
Type of BA						
I		2	(6.7%)	–		
II		1	(3.3%)	–		
III		27	(90.0%)	–		
Labo data						
AST	(IU/L)	170	(75–601)^a^	262	(19–825)^a^	0.451
ALT	(IU/L)	103	(37–375)^a^	217	(7–652)^a^	0.464
T-bil	(mg/dl)	8.2	(4.2–14.5)^a^	10.1	(7.3–14.7)^a^	0.268
D-bil	(mg/dl)	5.7	(2.6–11.0)^a^	4.2	(0.5–7.8)^a^	0.345
ALP	(IU/L)	1,848	(823–3,107)^a^	1,656	(495–2,431)^a^	0.841
GGTP	(IU/L)	670	(104–1,684)^a^	362	(45–1,253)^a^	0.0496^b^
TBA	(IU/L)	136.3	(83.6–272.3)^a^	–		–

BA, biliary atresia; AST, aspartate aminotransferase; ALT, alanine aminotransferase; T-bil, total bilirubin; D-bil, direct bilirubin;

ALP, alkaline phosphatase; GGTP, gamma glutamyl transpeptidase; TBA, total bile acid.

^a^ Median (minimum–max).

^b^ *p*-value < 0.05 by Mann–Whitney *U* test.

### 3.2 Multiple immunohistochemistry with pseudocolored images

**Figure 1:**
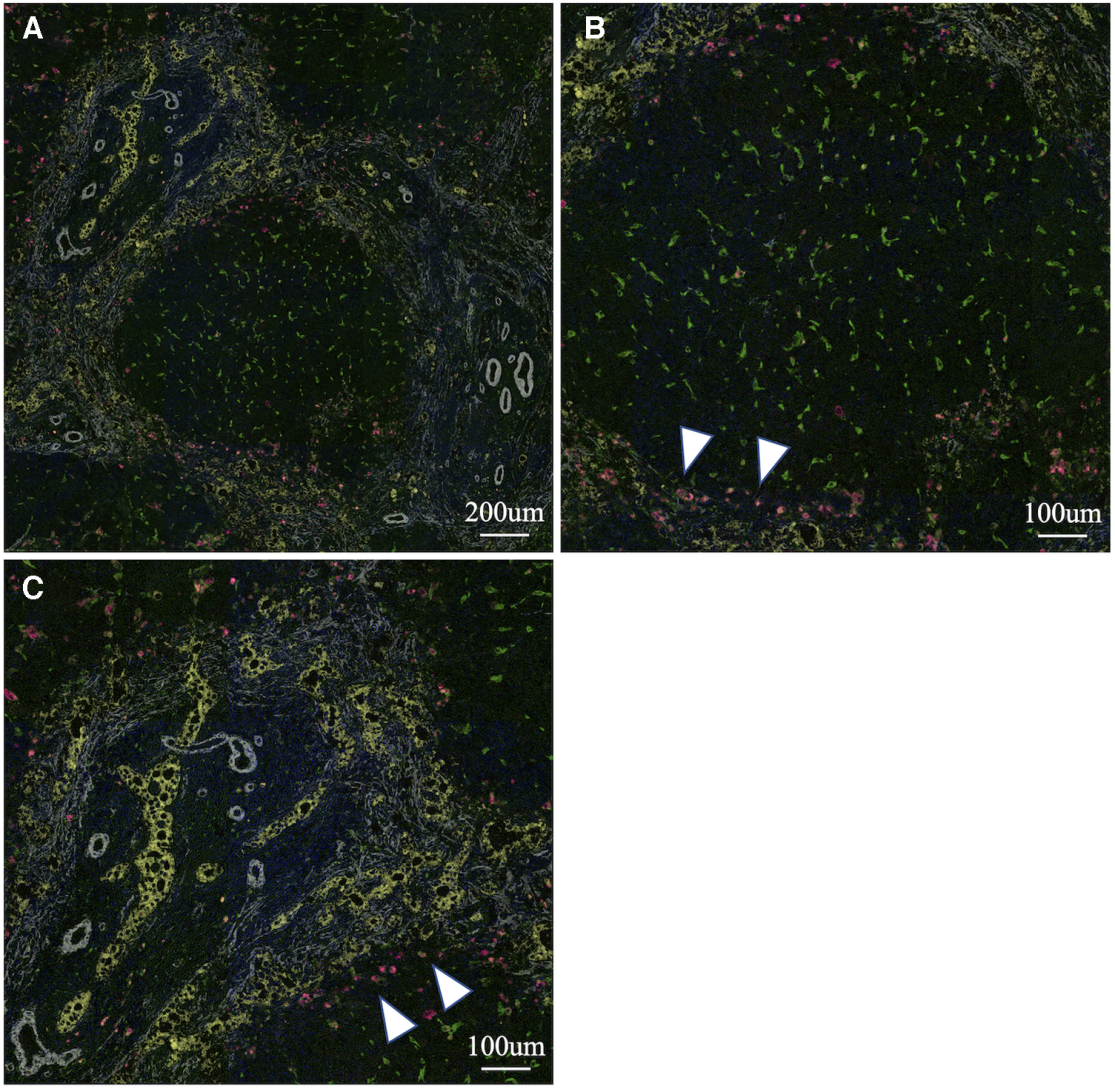
Multiple immunohistochemistry with pseudocolored images (non-M2: pink, M2: green, α-SMA: white, CK19: yellow). Samples of liver tissue obtained in patients with biliary atresia. A whole ROI image (**A**), a magnified image of the hepatic lobule (**B**), and a magnified image of the portal area (**C**). Non-M2 cells were mainly distributed along the portal area (white arrow) (finally assigned to M2 high group).

**Figure 2:**
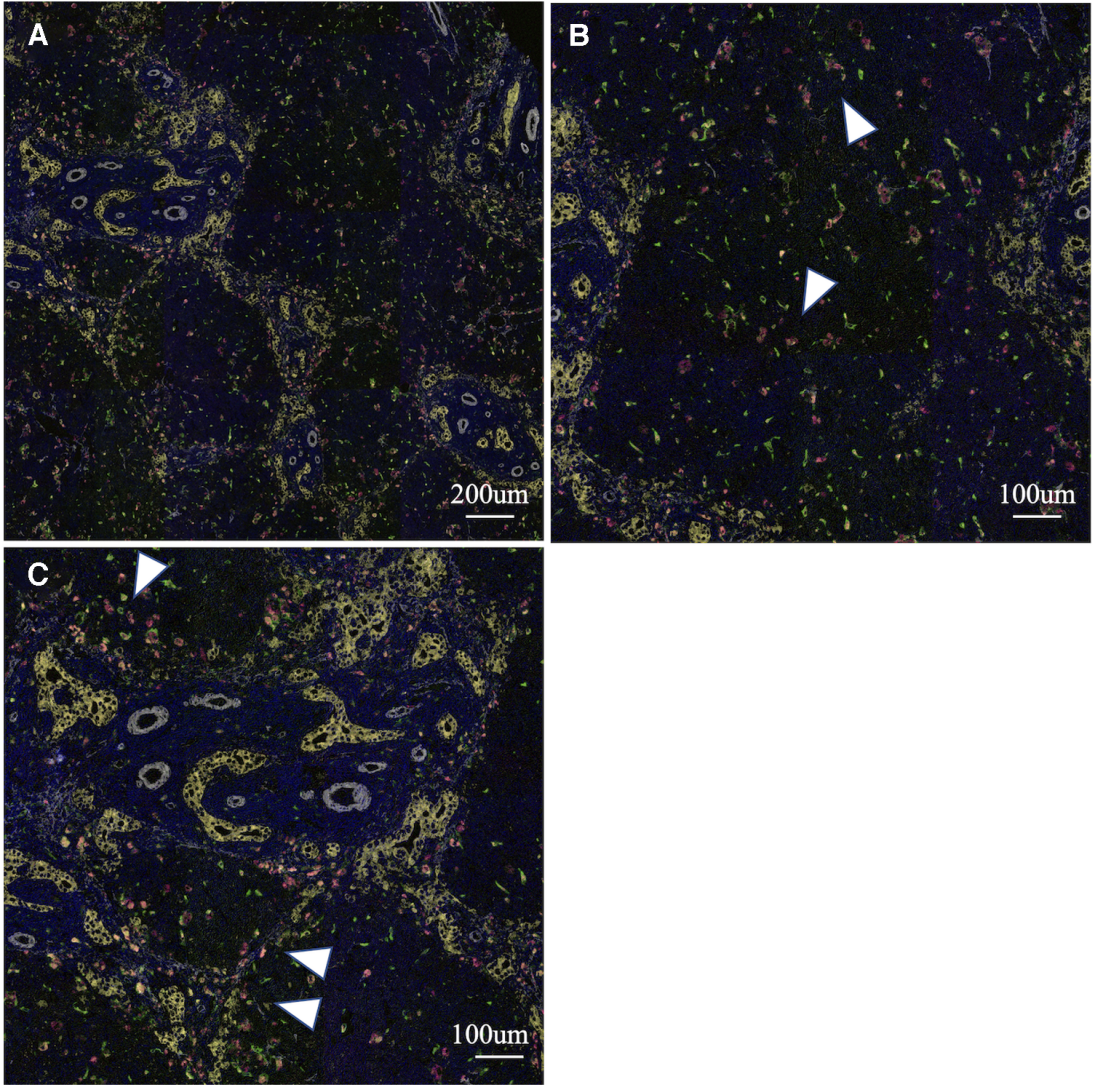
Multiple immunohistochemistry with pseudocolored images (non-M2: pink, M2: green, α-SMA: white, CK19: yellow). Samples of liver tissue obtained in patients with biliary atresia. A whole ROI image (**A**), a magnified image of the hepatic lobule (**B**), and a magnified image of the portal area (**C**). Non-M2 cells were also found widely in the hepatic lobule (white arrow) (finally assigned to M2 non-high group).

**Figure 3:**
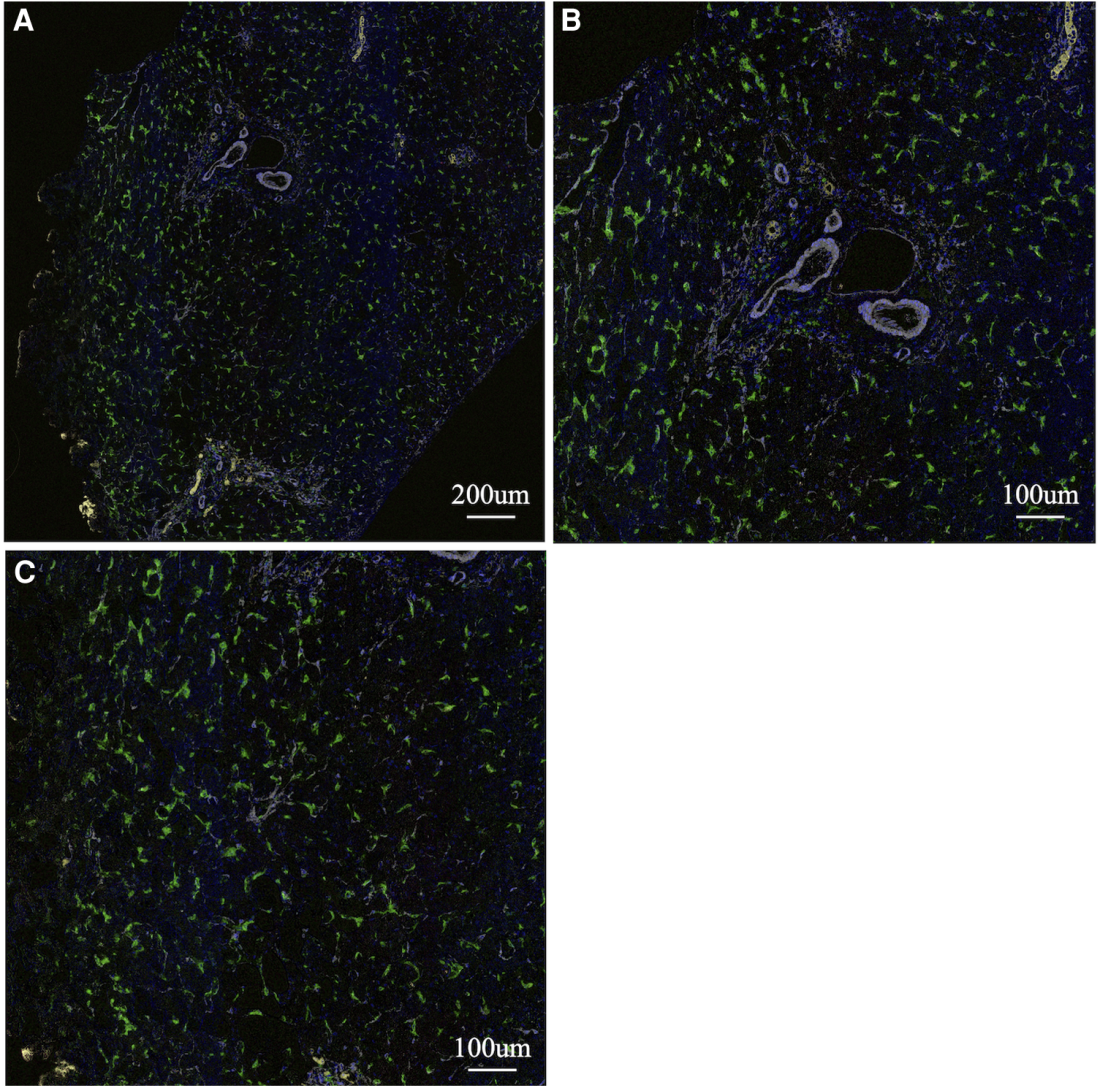
Multiple immunohistochemistry with pseudocolored images (non-M2: pink, M2: green, α-SMA: white, CK19: yellow). A representative of liver tissue in the control group. A whole ROI image (**A**), a magnified image of the hepatic lobule (**B**), and a magnified image of the portal area (**C**). There was no enlargement or fibrosis of the portal area, and almost all of the observed macrophages belonged to M2.

To analyse the cells involved in liver fibrosis quantitatively and visually, we created pseudocolored images as described above. From 35 cases (30 BA and 5 non-BA cases), 153 fields of view centered on the portal area were obtained. According to the above algorithm, we distinguished the macrophage phenotype, activated fibroblasts and proliferated cholangiocytes and assigned a color for each cell type (non-M2: pink, M2: green, α-SMA: white, CK19: yellow) (Figures 1–3). Figures 1, 2 were samples of liver tissue obtained in patients with biliary atresia, and Figure 3 was a representative of liver tissue in the control group. Each figure was consisted of a whole ROI image (a), a magnified image of the hepatic lobule (b), and a magnified image of the portal area (c). In Figure 1, non-M2 cells were mainly distributed along the portal area. In Figure 2, non-M2 cells were also found widely in the hepatic lobule. In Figure 3, there was no enlargement or fibrosis of the portal area, and almost all of the observed macrophages belonged to M2.

### 3.3 Macrophage polarity predicts the postoperative clinical course

**Figure 4:**
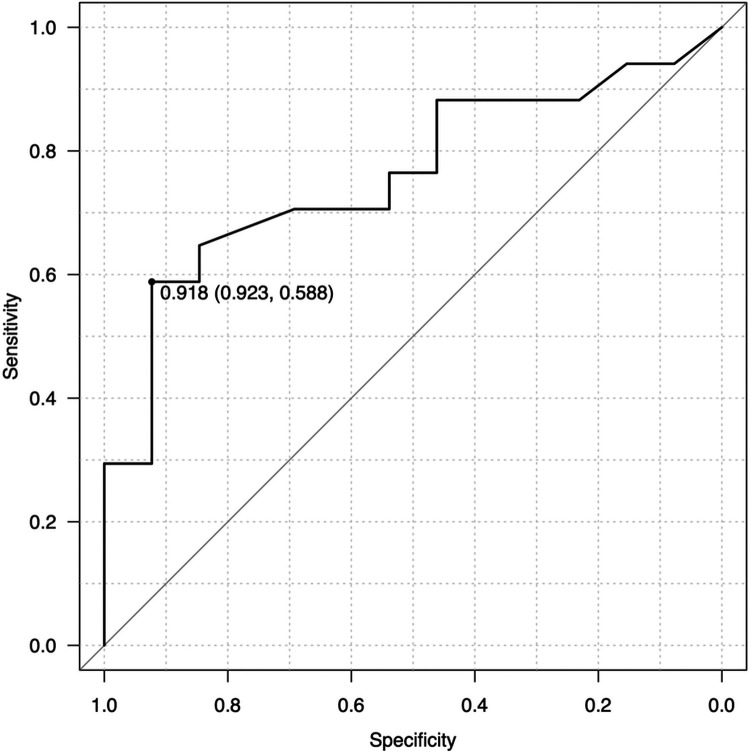
ROC analysis for the M2 ratio and postoperative cholangitis. The ROC curve calculated that AUC was 0.762 and Youden index was 0.918. As a predictor for cholangitis, the M2 ratio had a sensitivity of 58.8% and specificity of 92.3% when the cut-off value was set to 0.918.

Postoperative clinical courses for jaundice clearance, KPE redo, cholangitis, portal hypertension, thrombocytopenia, and liver transplantation were reviewed retrospectively (Table 2). Postoperative cholangitis was defined as illness with a fever not attributed to other sources and treated with antibiotics. Portal hypertension and thrombocytopenia were defined according to the diagnosis and treatment guidelines for aberrant portal hemodynamics by a Japanese study group (18). To analyse the correlation between the M2 ratio and postoperative clinical events, we generated ROCs with AUCs of 0.7 or greater as moderate accuracy. The AUC for the M2 ratio and postoperative cholangitis was 0.762, although the AUCs for other clinical results were less than 0.7. When we set a cut-off value of the M2 ratio to 0.918 according to the Youden index, postoperative cholangitis could be predicted with a sensitivity of 58.8% and specificity of 92.3% (Figure 4). Based on the M2 ratio, the patients with a ratio higher than 0.918 were categorized as the M2 high group (*n* = 19), and the others were categorized as the M2 non-high group (*n* = 11).

**Table 2:** Relationship between macrophage polarity (M2 ratio) and postoperative clinical course.

	Biliary atresia patients *n* = 30		AUC		Yauden index	*p*-value
Clearance of jaundice	26	(86.7%)	0.596	[0.364–0.828]^a^	0.935	0.29
KPE redo	7	(23.3%)	0.634	[0.410–0.857]^a^	0.923	0.372
Cholangitis	20	(66.7%)	0.760	[0.583–0.937]^a^	0.918	0.007^b^
Portal hypertension	16	(53.3%)	0.587	[0.374–0.800]^a^	0.958	0.928
Thrombocytopenia	12	(40.0%)	0.567	[0.345–0.790]^a^	0.976	1
Liver transplantation	11	(36.6%)	0.624	[0.409–0.840]^a^	0.935	0.266

KPE, Kasai portoenterostomy.

^a^ [95% CI].

^b^ *p*-value < 0.05 by chi-square test.

.

### 3.4 Comparison of pathological findings among the M2 high group, nonhigh group and non-BA group

**Figure 5:**
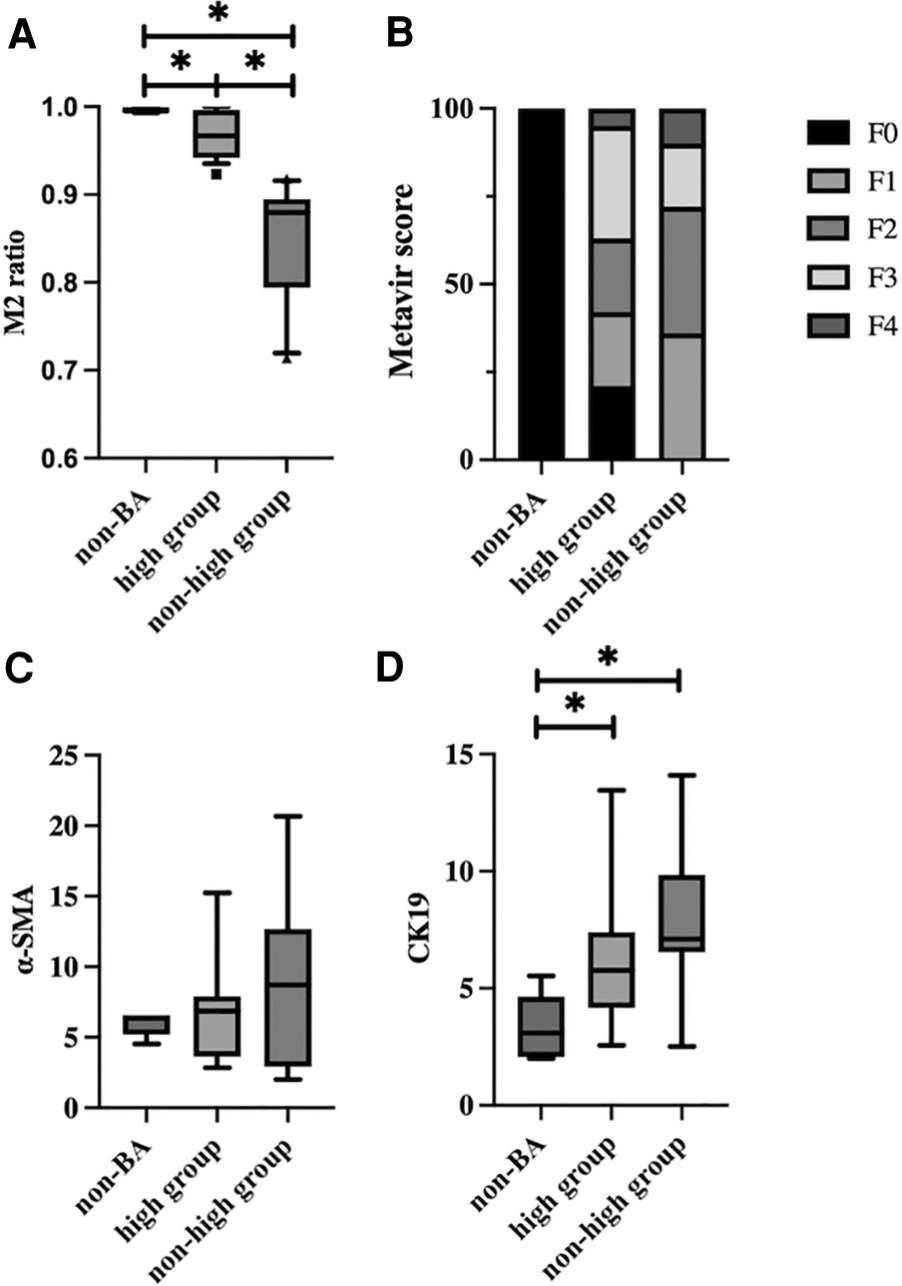
Comparison of pathological findings among the M2 high group, non-high group and non-BA group. (**A**) The M2 ratio for each group is shown. The M2 ratio was 0.995 (IQR 0.993–0.999) in the non-BA group, 0.967 (IQR 0.946–0.993) in the M2 high group, and 0.871 (IQR 0.786–0.893) in the non-high group. There were significant differences between each group (non-BA vs. M2 high, *p *= 0.0388; high vs. non-high, *p *= 0.000023; non-BA vs. non-high, *p *= 0.0044, respectively). (**B**) The percentage of Metavir fibrosis score for each group is shown. Although the Metavir fibrosis score did not show significant differences between the M2 high and non-high groups (*p *= 0.415), all five cases in the non-BA group (100.0%) and these cases in the M2 high group (15.8%) had no liver fibrosis. (**C**) The area fraction of α-SMA-positive cells per portal area for each group is shown. There was no significant difference among these 3 groups (*p *= 0.929). (**D**) The area fraction of CK19-positive cells per portal area for each group is shown. CK19-positive bile duct proliferation was significantly more often seen in the M2 high and non-high groups than in the non-BA group (*p *= 0.008), while no significant difference was noted between the M2 high and non-high groups (*p *= 0.278).

We compared histopathological findings among the M2 high group, non-high group and non-BA group. The mean M2 ratio was 0.995 (IQR 0.993–0.999) in the non-BA group, 0.967 (IQR 0.946–0.993) in the M2 high group, and 0.871 (IQR 0.786–0.893) in the non-high group (Figure 5A). Each group had significant differences in the mean M2 ratio (non-BA vs. M2 high, *p *= 0.0388; high vs. non-high, *p *= 0.000023; non-BA vs. non-high, *p *= 0.0044, respectively). Although the Metavir fibrosis score did not show significant differences between the M2 high and non-high groups (*p *= 0.415) (Figure 5B), all five cases in the non-BA group (100.0%) and three cases in the M2 high group (15.8%) had no liver fibrosis. The area fraction of α-SMA per portal area is shown in Figure 5C, and there was no significant difference among these 3 groups (*p *= 0.929). The area fraction of CK19 per portal area is shown in Figure 5D, and CK19-positive bile duct proliferation was significantly more often seen in the M2 high and nonhigh groups than in the non-BA group (*p *= 0.008), whereas no significant difference was observed between the M2 high and nonhigh groups (*p *= 0.278).

### 3.5 Impact of the M2 ratio on native liver survival rate

**Figure 6:**
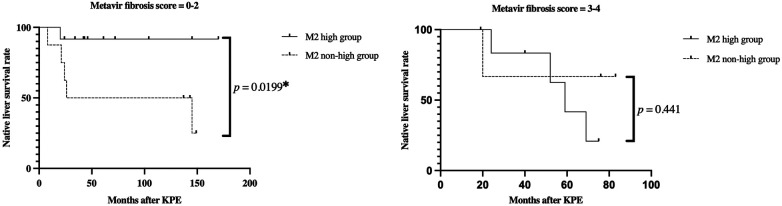
Kaplan-Meier curves for native liver survival rates in the M2 high and non-high groups. (**A**) Mild fibrosis cases (Metavir fibrosis score = 0–2) (**B**) severe fibrosis cases (Metavir fibrosis score = 3–4) in mild fibrosis cases, the M2 high group had a significantly higher native liver survival ratio than the non-high group (*p *= 0.0199). On the other hand, in the severe fibrosis group, there was no significant difference between the two groups (*p *= 0.441).

Native liver survival was analysed based on the degree of fibrosis at KPE. In patients with mild fibrosis (Metavir fibrosis score = 0–2), native liver survival in the M2 high group was significantly higher than that in the non-high group (*p *= 0.0199) (Figure 6A). In particular, early transplantation within 2 years after KPE was seen in four out of eight cases with non-high M2. On the other hand, there was no significant difference in native liver survival between the M2 high and non-high groups in patients with severe fibrosis (Metavir fibrosis score = 3–4) (*p *= 0.441) (Figure 6B).

## 4 Discussion

Since Kasai portoenterostomy was first reported in 1959, some patients with BA can obtain long-term survival with their native livers (19). The Japanese domestic registry reported that the 10-year native liver survival rate of BA was 53.2% (17). Despite jaundice clearance following successful KPE surgery, aggravation of liver fibrosis is seen in most patients. The current therapeutic option for advanced liver cirrhosis in BA is liver transplantation only, which demands a physical and psychiatric burden for patients and their families. Therefore, it is essential to elucidate the pathogenesis of fibrosis in the extrahepatic bile duct and liver parenchyma for the development of new noninvasive treatments for BA in terms of preventing liver fibrosis progression. Our previous study featured the relationship between IL-13 and periostin to reveal the etiology of extrahepatic bile duct obstruction (2). In this study, we focused on clarifying progressive fibrosis in the liver parenchyma.

Several studies have found macrophages to be involved in BA (6, 10, 15). The relationship between the infiltration of inflammatory cells into the portal area, including macrophages, and the surgical prognosis was reported by Davenport M et al. in 2001 (6), while the literature on macrophage polarity in BA has rarely been published. In this study, we analyzed the relationship between macrophage polarity and clinical results, and the ROC revealed that the M2 ratio might be a highly specific predictor for postoperative cholangitis with a sensitivity of 58.8% and specificity of 92.3%. This fact implied that cholangitis after KPE is more common in the group with a relatively high number of proinflammatory M1 phenotypes ( = M2 non-high group). In 2017, Yang Y et al. reported that BA cases with a high M2 ratio on the center of the hepatic lobules had high serum bilirubin levels and advanced liver fibrosis at KPE, and their limitation was the use of simple biomarkers to verify the polarity of macrophages, which applied to our study as well (4). Our study seemed contradictory to the results of Yang' study, and we speculated that differences in the region of interest in liver may have led to the discrepancy in the results.

Although there was no significant correlation between age at surgery and macrophage polarity (data not shown), in terms of the degree of liver fibrosis, the native liver survival rate of the M2 non-high group was significantly lower than that of the high group in mild fibrosis cases (Metavir fibrosis score = 0–2). Furthermore, early transplantation cases within 2 years after KPE were seen more often in the M2 non-high group. These findings implied that the progression of liver fibrosis can be slowed by regulating macrophage polarization for mild fibrosis cases, which may be a possible novel therapeutic target. Several macrophage-targeted attempts to improve or prevent liver fibrosis have recently been reported, including dexamethasone-induced polarization into antifibrotic macrophages in murine models and galectin-3 inhibitors under clinical investigation (3, 20). It has been pointed out that macrophage polarity may be more complex, and there is controversy regarding whether these findings of macrophage polarity are the cause or the consequence of the pathology of BA. Tacke F mentioned that macrophage therapy was challenging because the functions of macrophage subsets required for treatment may be variable and depend on disease stage (21). Therefore, we need to know more details of the functional aspects of macrophages and the interaction of macrophage polarity with other immune cells.

The utility of using multiple immunostainings in this study lies in the fact that even past formalin-fixed specimens can theoretically be evaluated with a number of antibodies from a single section. This method would be useful in the analysis of rare diseases such as biliary atresia. However, the present study had several limitations. Multiple immunostainings required repeated heat-induced epitope retrieval, which could result in tissue damage and nonspecific antigen expression. Unfortunately, we used only two antibodies (CD68/163) to distinguish from phenotypes of macrophages and cannot analyze the functional aspects of macrophages. The examined liver specimens were obtained at a single time point (at KPE only), and the time-course change in macrophage polarity was not investigated. Previous studies have found no significant difference in the relationship between the degree of macrophage infiltration to the liver tissue at KPE and postoperative clinical outcomes, and the impact of liver histology at KPE on the postoperative clinical course remains a matter of debate (21). This small series was from a single institution, and it is desirable to accumulate more cases for detailed analysis.

## 5 Conclusions

Non-M2 macrophages, including M1 macrophages, may be correlated with postoperative cholangitis, and the M2 non-high group in mild liver fibrosis cases had a significantly lower native liver survival rate than the high group, requiring early liver transplantation in this study. Preventing advanced liver fibrosis is a key factor in improving native liver survival for BA patients, and liver macrophages may play important roles in liver homeostasis and the promotion of inflammation and fibrosis.

## Data Availability

The original contributions presented in the study are included in the article/Supplementary Material, further inquiries can be directed to the corresponding author.
